# Design and evaluation of an educational mobile program for liver transplant patients

**DOI:** 10.1186/s12913-023-09989-1

**Published:** 2023-09-09

**Authors:** Mostafa Langarizadeh, Fateme Moghbeli, Shamim Ahmadi, Mohammad Hossein Langarizadeh, Mohammadjavad Sayadi, Fatemeh Sarpourian, Seyed Ali Fatemi Aghda

**Affiliations:** 1 Department of Health Information Management, School of Health Management and Information Sciences, Iran University of Medical Sciences, Tehran, Iran; 2 Department of Health Information Technology, Varastegan Institute for Medical Sciences, Mashhad, Iran; 3 Department of Computer Sciences, Faculty of Mathematics Sciences, Shahid Beheshti University, Tehran, Iran; 4 Department of Computer Engineering, Technical and Vocational University (TVU), Tehran, Iran; 5 Department of Health Information Technology, School of Management and Medical Informatics, Shiraz University of Medical Sciences, Shiraz, Iran; 6 Research Center for Health Technology Assessment and Medical Informatics, School of Public Health, Shahid Sadoughi University of Medical Sciences, Yazd, Iran

**Keywords:** Liver transplant, Educational needs, Self-care, Usability evaluation, Mobile phone health (mHealth)

## Abstract

**Background:**

Liver transplantation, the last treatment for advanced liver failure, necessitates patient education due to its wide range of complications and subsequent disabilities. The present study was development-applied research and aimed to design a mobile-based educational program to provide liver transplant patients with critical health information.

**Methods:**

In the first phase of the study, the crucial educational components were collected from the literature and organized in the form of a questionnaire using library studies and available global guidelines. The validity and reliability of this researcher-made questionnaire were confirmed by a panel of experts (*n* = 15), including gastroenterologists and liver specialists working in the Motahari liver clinic and AbuAli Sina Hospital in Shiraz. The application was designed followed by analyzing the data gathered from the first phase. To evaluate the mobile phone program’s usability, to evaluate the application, 30 liver transplant patients were randomly selected.

**Results:**

Most educational components covered in the questionnaire were deemed necessary by experts in the first phase. As a result, the educational contents were classified under 10 categories. The application had a good level of usability since the participants’ satisfaction score was 8.1 (out of 9 points).

**Conclusions:**

Due to the increase in liver transplantation and the use of mobile phones, applications increase the patient’s role in their health, and their awareness. It also leads to a better interaction and follow-up of the patient, the treatment staff of the medical centers.

## Introduction

Nowadays, the prevalence of chronic diseases is increasing at an exponential rate. This not only imposes negative impacts on people’s quality of life but also increases health care costs. Chronic liver disease, as a serious progressive condition of liver function [1], is one of the main global causes of death causing a wide range of diseases from different or unknown sources. This disease has been directly responsible for two to four% of all annual deaths throughout the world [2]. The literature is replete with studies conducted on the causes of advanced chronic liver failure in different parts of the world, according to the patients’ geographic region, race, lifestyle, age, and other variables. The results showed a discrepancy in findings regarding common causes of the disease in developing and high-income countries [3–7].

Liver transplantation is known as the last treatment for patients with chronic liver failure. In the last decade, the number of people waiting to receive a liver transplant has been increasing [8]. Although patients who undergo liver transplantation seek a better quality of life in the long term, they need intensive medical care after the operation that exposes them to various challenges, such as physical problems, fatigue, activity limitation, poor sleep, mental problems, as well as increased levels of stress, anxiety, and depression [9–11].

Lack of adherence to medications in transplanted patients, reflexes a serious problem leading to an increase in the rates of graft rejection and transplanted organ rejection [12]. Estimates indicate that half of the organ rejections and 15% of the transplant rejections are due to the lack of acceptance and continued use of immunosuppressive medicine [13, 14]. Since even small changes in the immunosuppressive regimen may be associated with poor outcomes and failure in transplantations, timely patient training is of great importance. Given that no adherence to medications is a complex and multifactorial problem, a combination of approaches is highly recommended targeting at different risk factors and building collaboration between patients and medical teams [15, 16].

To this end, most pre- and post-transplant care services usually take place in advanced centers far away from patients causing transportation, social, and economic challenges for the patients and their families [17–19]. The term ‘mHealth’ refers to the use of smartphones or digital devices for training, managing, monitoring, diagnosing, and treating diseases [20].

During the last decade, advancements in using computers, telemedicine, and mobile health (mHealth) applications, especially in drug evaluation, have affected many areas of healthcare, including transplantation [21]. On the one hand, the adoption of mHealth solutions is critical in providing knowledge and information to patients and health care providers. On the other hand, applications based on mobile phones are suitable due to their ease of access, acceptability, and support for social distancing, especially during the current COVID-19 epidemic [18, 22]. They also provide innovative solutions for disease management, hope for patients, and help for caregivers and health professionals. These applications cover a wide range of clinical knowledge that can be used by healthcare professionals for specific diseases/disorders in patient-centered approaches [23, 24].

Most of the published telemedicine literature in the field of liver transplant disease is aimed at disease management and improvement of care [25–27]. These scholars noted distance education as a suitable solution for providing patients with advice and supporting them.

The survival rate of patients after receiving a transplant depends on post-transplant care, which requires adequate training of the patient in various fields, such as diet, drug regimen, infectious disease education, daily activities, and physical activities. Education is necessarily needed for liver transplant recipients to promote their health and participate in their own treatment. Educational programs improve health behaviors, effective self-care, symptom management, and control of other diseases caused by liver transplantation [28]. In this regard, information needs analysis and educational contents required by liver transplant recipients are among the main prerequisites for providing proper self-management training by health care providers [11, 29]. As a result, levels of successful transplantation, survival, and quality of life increase. Considering the lack of self-care educational software based on the people’s geographical location, race, lifestyle, etc. in Iran, designing and evaluating a mobile phone-based training application seem necessary for liver transplant patients according to the users’ needs analysis and opinions of experts. This application can be useful in increasing the patients’ levels of awareness and satisfaction.

## Methods

### Study design

This quantitative study was carried out in two phases. In the first phase, a researcher-made questionnaire was developed and evaluated by a panel of experts who were required to determine the appropriate educational content for the application. In the second phase, the application was designed based on Android and its usability was evaluated by users.

To determine the appropriate educational contents and capabilities required for designing the application program, a list of educational contents and components was prepared followed by reviewing the literature and various studies in the form of a researcher-made questionnaire. Respondents, including 15 experts (selected based on the purposeful sampling method) were supposed to read the items and evaluate them as necessary (1 score) or unnecessary (0 score). This questionnaire consisted of three sections: participants’ demographic information (3 items), educational contents (in 10 general categories and a total of 57 items), and the required capabilities or skills (4 items).

The content validity of the questionnaire was confirmed by six faculty members of medical informatics, gastroenterology, and liver specialty. The questionnaire’s reliability was also confirmed by gastroenterologists and liver specialists using the Kuder-Richardson method (KR-20 = 0.85). After completing the questionnaire, the results were analyzed using descriptive statistics and frequency distribution reports. The educational contents were included in the program in the case that the related items were approved by 65% ​​of the experts.

The application was designed based on the educational content collected from the first phase. This application was developed using Visual Basic programming language, Java Class Library, and in the B4A environment. To evaluate the application’s usability, it was installed on the smartphones of Liver transplant patients (*n* = 30) referring to Motahari Clinic and Abu Ali Sina Hospital in Shiraz affiliated with Shiraz University of Medical Sciences from May to June 2022. Prior to installing the applications, oral consent was obtained from all participants after providing them with comprehensive explanations of the study goals. Patients were selected based on the number of available cases and their willingness to cooperate in the intervention. Other inclusion criteria were having a smartphone with Android version 5 and higher and having had the transplantation for at least one month. Two weeks after the patients used the application, they were asked to evaluate its usability by the standard Quiz version 5.5 questionnaire. The validity and reliability of the questionnaire have already been confirmed by previous studies. The evaluation section of the questionnaire included: general opinion about using the application (6 items), its screen (4 items), terminology and information (6 items), learnability (6 items), and general capabilities (5 items). Each item was responded based on a 10-point Likert scale from zero (lowest) to nine (highest).

## Results

Followed by reviewing the related literature, a questionnaire was developed on educational content required for designing a mobile-based application for patients with liver transplantation. The questionnaire was administered to 15 gastroenterologists and liver specialists who were required to determine the necessary concepts to be covered by the application. The experts’ demographic characteristics (Table 1) showed that most participants were male, were within the age range of 40 to 50 years, and had more than 10 years of work experience.

The participants were asked to read the questionnaire’s items carefully and determine the educational content required for developing the application program. Table 2 represents the experts’ viewpoints in this vein.

**Table 1:** Frequency distribution of experts’ demographic characteristics

Variable	n	%
Gender		
Male	12	80
Female	3	20
Age		
< 40	1	6.7
40–50	10	67
> 50	4	26.3
Work Experience		
5–10	1	6.7
11–20	9	60
> 20	5	33.3

**Table 2:** Frequency distribution of experts’ responses regarding the necessity of educational items

Row	Category	Educational items	AGREE	Row	Category	Educational items	AGREE
1	**Transplantation medicine**	Importance of transplantation medicine	100	29	**Gastrointestinal complications after liver transplantation**	Digestion of food	67
2		Cyclosporine	100	30		Causes of digestive disorders	93
3		Tacrolimus	100	31		Treatment of gastrointestinal complications	73.4
4		Mycophenolate mofetil	100	32		diarrhea	67
5		Prednisolone and azathioprine	100	33		Symptoms of peptic ulcer	73.4
6		Important points in the using medicine	93	34		Prevention of wounds	93
7	**Diet**	Fats	100	35		Inflammation of the digestive tract	80
8		Carbohydrates	100	36		Swallowing stimulants and spicy substances	67
9		Vitamins	100	37		Esophagitis in transplant recipients	80
10		Minerals	86.7	38		Gastritis	73.4
11		Proteins	86.7	39		Pancreatitis	73.4
12		Fruits and vegetables	80	40	**Diabetes**	About diabetes	100
13		Milk and diaries	80	41		Post-transplant diabetes	100
14		Oils	80	42		Reduced risk of diabetes	93
15		Liquids	60	43		Signs and symptoms of diabetes after transplantation	93
16		Important recommendations	86.7	44		Complications of Diabetes	100
17	A**ctivities**	Kinds of sports	53.4	45		Living with diabetes	93
18		Starting exercise after transplantation	86.7	46	**Oral care**	Oral care	86.7
19		Increasing the level of activity	86.7	47		The most common dental problems in transplant recipients	93
20		Appropriate exercises	100	48		Correct brushing	80
21		Important recommendations	93	49		Performing dental treatments after transplantation	67
22	**Skin Cancer**	Skin cancer cases	80	50	**Motherhood**	Transplantation and pregnancy	93
23		Effective factors	73.4	51		The best time of pregnancy	100
24		Treatment	73.4	52		Pregnancy complications	93
25	**Cytomegalovirus**	About cytomegalovirus	86.7	53		Pregnancy risks for the fetus	86.7
26		Diagnosis of cytomegalovirus	93	54		Lactation	93
27		Symptoms of cytomegalovirus	93	55	**Sexual desires**	Sexual desires	93
28		Treatment of cytomegalovirus	86.7	56		Initiation of sexual activity after transplantation	93
				57		Decreased libido	93

As illustrated in Table 2, the obtained educational components were classified under 10 categories include: transplantation medicine (6 items), nutrition (10 items), exercise (5 items), skin cancer (3 items), cytomegalovirus (4 items), gastrointestinal complications after liver transplant (11 items), diabetes (6 items), oral and dental care (4 items), motherhood (5 items), and sexual desire (3 items). The majority of items were approved by the experts and included in the application. However, liquids (in the nutrition category) and types of exercise (in the activities category) were deemed unnecessary by the experts. The experts’ opinions about the application capabilities are listed in Table 3.

An open-ended question was also added to the end of the questionnaire asking the participants to add their possible opinions and suggestions not covered in the questionnaire. After collecting and summarizing the information, 50% of the experts believed that” body mass index (BMI) calculation” and “medication reminders” were among the most significant features of the application design.

Followed by determining the required educational items, a mobile phone-based application was designed and installed on the mobile phones of 30 liver transplant patients who referred to Motahari Clinic and Abu Ali Sina Transplantation Hospital of Shiraz affiliated with Shiraz University of Medical Sciences. To observe ethical considerations, verbal consent was obtained from all patients prior to the intervention. Demographic information of the participants included gender, age, education level, and Duration after the transplant operation (Table 4).

**Table 3:** Distribution of the experts’ answers regarding the application’s capabilities

Capabilities	Necessary	
	n	%
Customizability For the user	12	80
Menus for quick access to content	12	80
The ability to play videos and animations by the user	13	86.7
Introducing digital education resources to the user	14	93.3

**Table 4:** Frequency distribution of the participants’ personal characteristics

Variable	n	%
Age		
< 40	9	30
40–50	17	56.7
> 50	4	13.3
Gender		
Male	22	73.3
Female	8	26.7
Duration after the transplant operation		
Less than a year	10	33.3
1–2 years	13	43.3
More than 2 years	7	23.4
Education level		
High school	2	6.7
Diploma	5	16.6
Associate Degree	3	10
Masters	8	26.7
Masters and above	12	40

The majorities of participants were Male, had since their transplant for more than a year, and were in the age range of 40 to 50 years. Considering their level of education, about 66.7% (*n* = 20) had a Master’s degree or higher. The users’ evaluations and.

opinions regarding usability of the designed application were collected after two weeks (Table 5).

**Table 5:** The end-users’ evaluation of the usability of the application

Scale	Mean	SD
Overall Reactions To The Software	8.1	0.64
Screen	7.6	0.93
Terminology And System Information	8.45	0.41
Learning	7.9	0.96
System Capabilities	7.5	0.84
Usability And User Interface	7.9	0.75

## Discussion

Given the current need to increase patients’ self-care awareness, especially those with chronic diseases, mHealth applications can be employed to reduce costs and enhance the availability of treatment services [30–32]. The present study aimed at designing and evaluating an educational application for liver transplant patients. To this end, the necessary parameters and educational contents were classified under 10 categories of transplantation medicine, nutrition, daily activities, gastrointestinal complications after liver transplantation, diabetes, skin cancer, megalovirus, oral care, motherhood, and sexual desire were obtained based on the library studies and experts’ opinions. The evaluation results indicated that the application was at a good level of usability with an average score of 7.9 ± 0.75.

In this regard, Wadhwani et al. [33]. Mentioned the medical and social needs of liver transplant recipient children and noted some solutions to improve the care process. The participants, who were parents and caregivers of children, were asked to answer questions about the liver transplant recipient children in an interview. According to the findings, the caregivers were at high levels of motivation and ability regarding the transplantation. The participants also provided the research team with several suggestions to improve care, such as increasing the awareness of parents and children. These researchers concluded that the caregivers’ knowledge was not sufficient about post transplantation caring. Consequently, the present study was conducted to increase the awareness of parents and families with Liver transplant recipients.

Joyce et al. [34] carried out a six-month educational course to empower the patients using virtual education. As they reported, the intervention not only obtained positive feedback from the patients but also increased their awareness and participation. The participants maintained the significant role of technology, better support, development of a standard workflow, etc. Our study is in line with this study except that we administered the training program based on a mobile application and tried our best to achieve a high level of usability based on the end-users’ needs.

Considering the liver transplant recipients’ rapid weight gain and lack of exercise, Hickman et al. [35]. Applied remote health to change their lifestyle (including exercise and diet). Through a 12-week intervention via video telecast, 14 sessions were held by nutrition and exercise specialists for liver transplant recipients. Similar to our study, the post-intervention findings confirmed high levels of reliability and effectiveness for the program, reduced severity scores of metabolic syndromes, and improved diet adherence, quality of life, and mental health. However, all the required educational content and components (including nutrition) were collected through a literature review, corroborated by the experts via a questionnaire, administered among patients (as end users) via an application based on a mobile phone platform, and evaluated in terms of its usability.

Gordon et al. [36]. Evaluated the effectiveness of a web-based application entitled “Notify Me” to increase awareness among kidney recipients. Participants were randomly assigned to the control (regular training) and intervention (regular training along with using the “Notify Me” application) groups. The evaluation was performed in two phases using questionnaires and phone calls. A comparison between scores of the two study groups showed that members of the intervention group obtained a higher knowledge score after the intervention. In another study, Korus et al. [37]. Investigated the acceptance and effectiveness of a web-based self-care application designed for adolescent liver transplant candidates or recipients. The control group had no access to the application while the intervention group members were required to use the application. The evaluation criteria included the application’s feasibility and usage rate, health-related outcomes in self-care assessment, and knowledge level. Data were collected using a semi-structured interview after the intervention and the findings represented higher levels of motivation to practice and learn in the intervention group and patients who had received the transplantation less than a year before the data collection. The participants mentioned obstacles, such as slowness and lack of constant access to the program. As these researchers found, no significant difference was observed between the groups with regard to health-related outcomes. In the same vein, Wedd et al [38]. Investigated the use of portals in liver and kidney transplant recipients (*n* = 710) according to the patients’ specific needs at the Transplantation Center of Southeastern, United States. The portal had features, such as drug lists, laboratory results, secure messages, health records, etc. The findings showed the significant effect of educational conditions and race on the users’ application of the portal. Due to the portal’s efficiency, it has been recommended as a suitable tool for health management and patient support. In comparison with the above-mentioned studies, the present study employed a panel of experts to determine the appropriate training content, designed an application based on mobile phones, and evaluated usability of the application.

In a prospective cohort study, Koc et al. [39]. Examined the clinical feasibility, safety, and beneficial effects of a telemedicine-based tele monitoring program (TRMP) for continuous follow-up of adult liver transplant recipients. This study was conducted at the University Hospitals of Leuven, Belgium. The findings did not indicate any increase in the rate of liver transplant rejection and the need for hospitalization caused by tele monitoring. Researchers reported the patients’ high levels of participation and willingness as well as stable clinical condition to run TRMP. This program could reduce the rate of outpatient visits and improve a safe approach. In other words, remote monitoring has provided the opportunity for accurate follow-up of tacrolimus levels. Unlike this study, we conducted an educational program based on the previous studies and experts’ opinions, tried to obtain all the important educational content, and provided the liver transplant recipients with these achievements in the form of an educational program.

In promoting the caring process for patients with chronic diseases, Hayward et al. [40]. Developed a questionnaire and administered it to cirrhosis patients to determine their level of information about liver disease and the required self-management measures. Half of the patients remembered the written information provided by the clinical expert and 64% reported that obtained the information personally, mostly from the Internet. The educational pamphlet administered in this study had a simple structure, descriptive photos, and plain language to provide patients with their needed information between each follow-up period. Different from the mentioned study, we collected and represented all the post-transplantation information up to the time of recovery when patients returned to their previous lifestyle.

Among the limitations of this study, the following can be mentioned. Due to the epidemic of COVID-19 during the study period, the specialists participating in this research were selected from one of the specialized liver transplant hospitals in Iran. In some cases, experts did not complete the questionnaires comprehensively due to their busy schedules and/or personal lack of motivation. To meet this lack of cooperation by experts, the researcher referred back to the specialist with an incomplete questionnaire, explained the study’s significance and purpose, and asked them to complete the questionnaire. Also, due to the aforementioned problems in accessing the participants, it was not possible to use a questionnaire for nutritional risk, so only parameters such as BMI were used. In this study, the questionnaire’s evaluation section only consisted of the application’s usability. Moreover, only the experts determined and confirmed the educational content needed for the application. To obtain more generalizable results, future researchers are suggested to include experts from the specialized transplantation centers in Iran and obtain the end users’ points of view along with the experts’ ideas to determine the required educational content.

## Conclusion

The present study aimed to design an educational mobile-based application for Liver transplant patients based on Android. As a result, the necessary educational content and required educational components were obtained and classified under 10 main categories using the experts’ points of view. The participants’ high evaluation scores showed that users were satisfied with the usability of this program. Regardless of the time and place limitations, this application could increase the patients’ awareness in the fields related to their disease, participation and involvement in their treatment process, and quality of care. Furthermore, this application was able to reduce complications of liver transplantation while increasing the public’s awareness.

## Data Availability

The data used and analysed during the current study are not publicly available due Iran University of Medical Sciences policy but are available from the corresponding author on reasonable request.
